# Sex-specific shape models to disentangle sex effects and age-associated anatomical variation in biventricular cardiac anatomy

**DOI:** 10.1093/ehjimp/qyag156

**Published:** 2026-09-07

**Authors:** Stefano Buoso, Valery L Visser, Pia Leuders, Tobias Kudelka, Maximilian Fuetterer, Christina Spengler, Robert Manka, Sebastian Kozerke

**Affiliations:** Institute for Biomedical Engineering, ETH and University of Zurich, Gloriastrasse 37/39, 8092 Zurich, Switzerland; Institute for Biomedical Engineering, ETH and University of Zurich, Gloriastrasse 37/39, 8092 Zurich, Switzerland; Institute for Biomedical Engineering, ETH and University of Zurich, Gloriastrasse 37/39, 8092 Zurich, Switzerland; Institute for Biomedical Engineering, ETH and University of Zurich, Gloriastrasse 37/39, 8092 Zurich, Switzerland; Institute of Diagnostic and Interventional Radiology, University Hospital Zurich, University of Zurich, Zurich, Switzerland; Institute for Biomedical Engineering, ETH and University of Zurich, Gloriastrasse 37/39, 8092 Zurich, Switzerland; Institute for Human Movement Sciences and Sport, ETH Zurich, Zurich, Switzerland; Institute of Diagnostic and Interventional Radiology, University Hospital Zurich, University of Zurich, Zurich, Switzerland; Department of Cardiology, University Hospital Zurich, University of Zurich, Zurich, Switzerland; Institute for Biomedical Engineering, ETH and University of Zurich, Gloriastrasse 37/39, 8092 Zurich, Switzerland

**Keywords:** population imaging phenotyping, age-associated anatomical variation, cardiovascular magnetic resonance, statistical shape modelling, biventricular models, sex-specific anatomical variation

## Abstract

**Aims:**

Age- and sex-related differences in cardiac anatomy are well established using conventional cardiovascular magnetic resonance biomarkers, but their representation in 3D ventricular shape remains unclear. In pooled statistical shape models, leading modes may be dominated by between-sex anatomical variation, potentially obscuring within-sex age-associated variation. This study evaluated whether sex-specific statistical shape models provide a more interpretable representation of age-associated ventricular variation than a sex-agnostic model.

**Methods:**

Biventricular meshes were reconstructed from cine cardiovascular magnetic resonance (CMR) images in a cross-sectional screened asymptomatic Swiss adult cohort aged 18-85 years. End-diastolic anatomies from 185 male and 187 female participants were used to construct statistical shape models for the pooled cohort and separately by sex. Shape modes were interpreted using anatomical visualization, variance decomposition, associations with conventional demographic, anthropometric, and CMR biomarkers.

**Results:**

The first pooled model mode explained 31% of total shape variance, compared with 22% and 19% for the first male- and female-specific modes. In the pooled model, 57% of first-mode variance was attributable to between-sex differences. Age-associated variation in the pooled model was distributed across the first two modes, whereas sex-specific models organized age-associated variation within each sex. In males, the strongest adjusted age association occurred in the second sex-specific mode, which correlated with ventricular volume and mass. In females, age-associated variation was concentrated in the first mode and was inversely associated with ventricular volumes and myocardial mass.

**Conclusion:**

These findings support sex-specific anatomical reference models for capturing within-sex cardiac ageing structure.

## Graphical Abstract

**Graphical Abstract:**
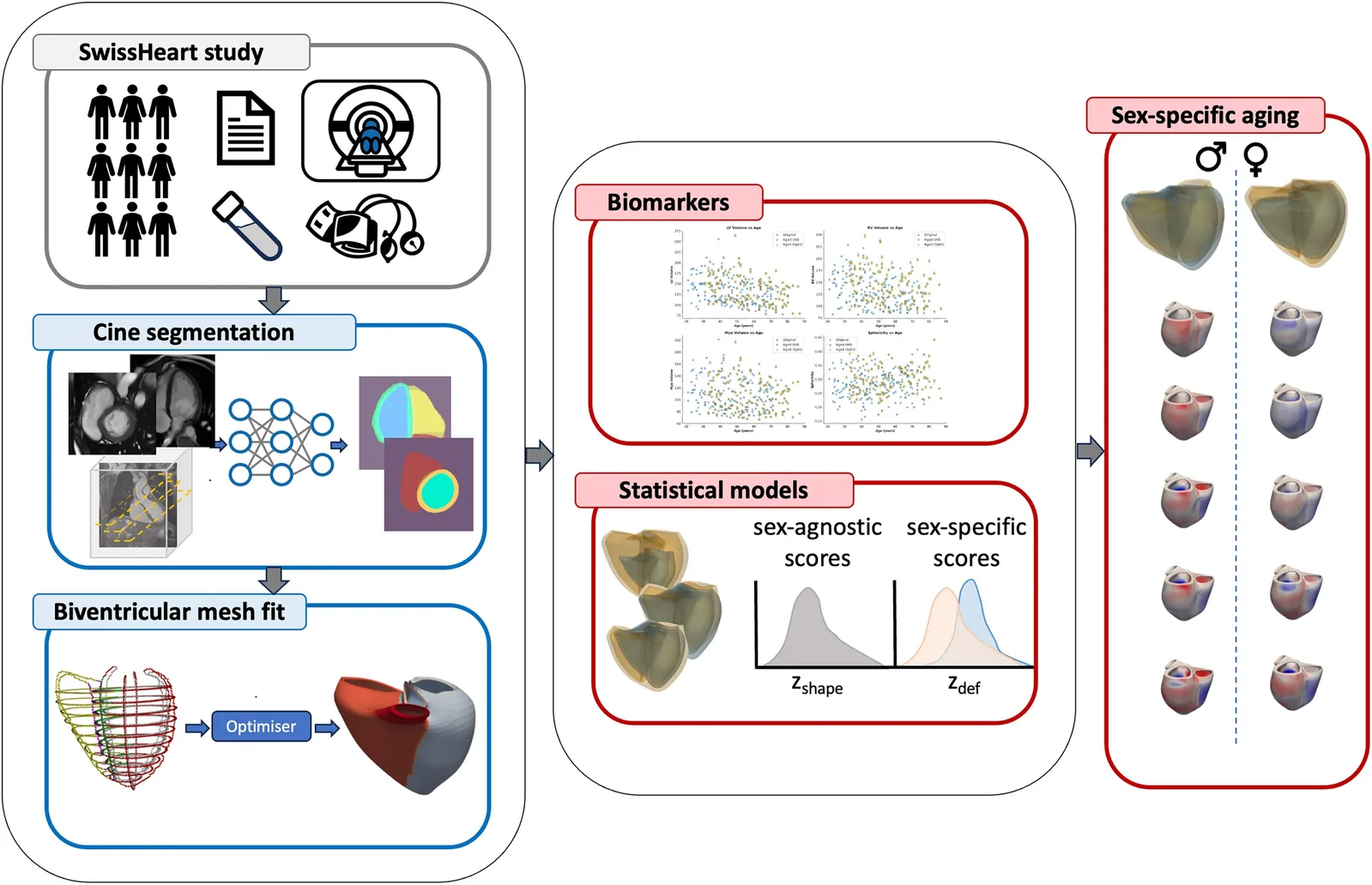

## Introduction

In cardiovascular magnetic resonance (CMR), cardiac function is commonly quantified using global indices such as ventricular volumes, ejection fraction (EF), myocardial strain, and valve annular motion.^1-3^ These biomarkers show consistent associations with age and sex^4-6^ but provide only a limited description of cardiac structure. Measures of ventricular geometry can offer complementary information by reflecting interactions between chamber form, wall stress, and afterload,^7-9^ and have been associated with adverse cardiovascular outcomes.^10-12^

Statistical shape modelling extends conventional CMR phenotyping by representing cardiac anatomy as a compact set of quantitative descriptors.^13^ Instead of reducing anatomy to a small number of predefined measurements, this approach captures coordinated patterns of three-dimensional variation across the ventricular surfaces. Shape-derived descriptors have been used to characterize physiological and pathological cardiac phenotypes and may capture anatomical information not fully represented by standard volumetric or functional biomarkers.^14^ With the increasing availability of large imaging cohorts such as the UK Biobank^15^ and Multi-Ethnic Study of Atherosclerosis (MESA),^16^ population-level studies have shown that shape-derived descriptors may capture aspects of cardiovascular risk that are not fully reflected by standard biomarkers.^14,17-20^

Despite well-established sex differences in cardiac size, function, and remodelling patterns, many shape-based analyses construct a single pooled anatomical model across males and females.^17,21^ In such sex-agnostic models, leading modes of variation may reflect global cardiac scale or between-sex differences in mean anatomy. This is not necessarily problematic when the objective is to describe total population variability, but it can complicate interpretation when the objective is to characterize anatomical variation within sex. In particular, if dominant pooled modes are driven by scale or sex-related anatomy, more specific within-sex age-associated patterns may be distributed across multiple components or assigned to lower-ranking modes.^21,22^

In parallel, although age-associated trends in conventional CMR biomarkers are well characterized^23-25^, the relationship between age and detailed three-dimensional ventricular form remains less clearly defined.^26,27^ Findings from MESA^28^ indicate that cross-sectional cohort-level trends and longitudinal biomarker trajectories may differ, partly because of variation in cardiovascular risk factor profiles. This highlights the need to establish reference patterns in the low-cardiovascular-risk population, both for conventional biomarkers and for shape-derived descriptors, to support more accurate interpretation of age-associated anatomical variations.

It also remains unclear how age-associated biventricular anatomy is represented when shape variability is modelled within sex rather than in a pooled population. A pooled model may provide an efficient description of total anatomical variance, but may conflate age-associated anatomy with global scale and between-sex differences. Conversely, sex-specific models may reorganize the same anatomical information into modes that better reflect within-sex variation and are therefore more directly interpretable in relation to age and conventional CMR biomarkers.

This study evaluated sex-agnostic and sex-specific statistical shape models of end-diastolic biventricular anatomy in a cross-sectional Swiss CMR cohort without manifest cardiovascular disease. We tested whether the leading modes of a pooled model were dominated by global scale and between-sex anatomical variation, and whether sex-specific models provided a more compact and interpretable representation of within-sex age-associated ventricular anatomy. Model interpretability was assessed using anatomical visualization, variance decomposition, associations with conventional CMR biomarkers, and multivariable age associations adjusted for body size and haemodynamic covariates.

## Methods

*Figure 1* summarizes the analysis workflow. Cine cardiovascular magnetic resonance images from the SwissHeart cohort were segmented automatically and used to fit subject-specific biventricular meshes across the cardiac cycle. End-diastolic biventricular meshes were then used to construct statistical shape models for the pooled cohort and separately for males and females. The resulting anatomical scores were analysed in relation to age, conventional CMR biomarkers, and anthropometric and haemodynamic covariates.

**Figure 1:**
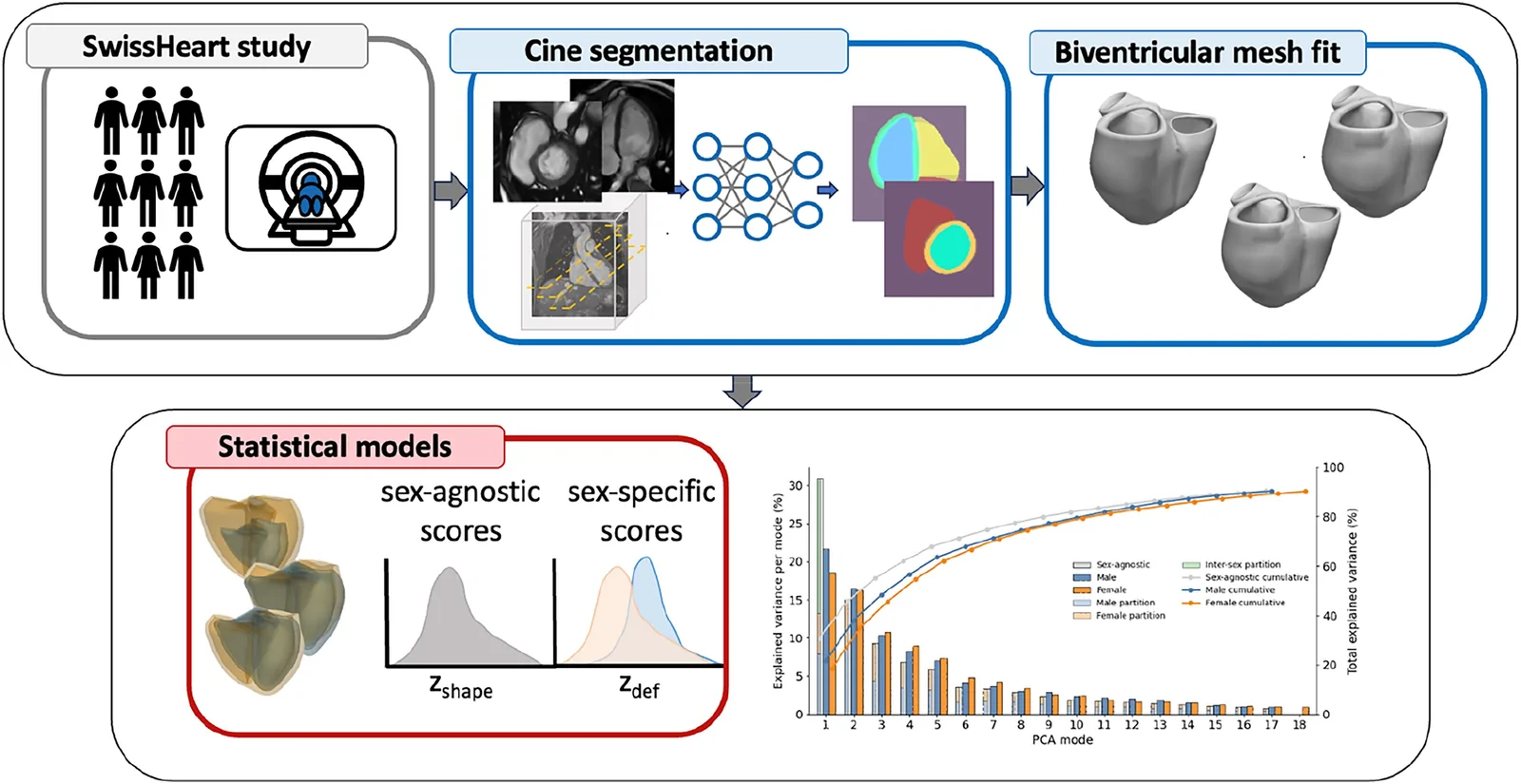
Overview of the analysis framework. Input data comprised CMR images from 382 volunteers from the SwissHeart study. Long- and short-axis cine images were segmented using nnU-Net models.^29^ Three-dimensional thorax scans were resampled to cine planes, segmented with the same networks, and used to correct for breath-hold-induced offsets. Segmentation contours were then employed to fit a biventricular mesh at each cardiac frame. The resulting mesh population was used to derive conventional cardiac biomarkers, as well as anatomical sex-agnostic and sex-specific statistical shape models.

### Dataset

This study used data from the SwissHeart cohort, a cross-sectional CMR study of asymptomatic adult volunteers aged 18–85 years. All participants provided written informed consent, and the study was approved by the Ethics Committee of the Canton of Zurich, Switzerland.

Imaging was performed on a 1.5 T MR scanner (Philips Healthcare, Best, The Netherlands). Balanced steady-state free precession (bSSFP) cine images were acquired using a short-axis stack covering the entire heart and three long-axis planes (two-, three-, and four-chamber) at a spatial resolution of 1.6 × 1.6 mm^2^ (reconstructed to 1.0 × 1.0 mm^2^) with 8 mm slice thickness, flip angle 60°, TR/TE 2.6/1.3 ms, temporal resolution 44 ms. Additionally, a respiratory-gated three-dimensional whole-thorax bSSFP scan was acquired in late diastole (acquired/reconstructed spatial resolution: 1.8 × 1.8 × 1.8 mm^3^/0.8 × 0.8 × 0.8 mm^3^) to provide anatomical context for registration. Brachial peak systolic and diastolic blood pressures and resting heart rate were measured prior to the CMR examination.

The present analysis included participants of Caucasian/White ethnicity without manifest cardiovascular disease. Manifest cardiovascular disease was defined as a history of myocardial infarction, coronary artery disease, arrhythmia, myocardial hypertrophy, aortic dissection, aortic valve stenosis, cardiomyopathy, atrial fibrillation, valve regurgitation, or hypertension. A total of 193 males and 189 females met these criteria. After exclusion of 10 participants because of automated segmentation or mesh-fitting failure, the final analysis cohort comprised 372 participants, including 185 males and 187 females. *Table 1* describes the demographic, anthropometric, and haemodynamic characteristics of the SwissHeart volunteers included in the final analysis cohort (the full cohort characteristics are reported in the Supplementary data online, *Table A1*).

**Table 1:** Demographic, anthropometric, haemodynamic and clinical characteristics of the SwissHeart volunteers included in the final analysis cohort

Characteristics	Males (*n* = 185)	Females (*n* = 187)
Demographics		
Age, years, mean (SD)	46 (15)	46 (15)
Anthropometrics		
Weight, kg, mean (SD)	81 (12)	63 (9)
Height, cm, mean (SD)	180 (7)	166 (7)
BSA, m^2^, mean (SD)	2.01 (0.16)	1.70 (0.12)
Haemodynamic		
SBP, mmHg, mean (SD)	132 (15)	120 (16)
DBP, mmHg, mean (SD)	80 (10)	79 (11)
Clinical characteristics		
Diabetes, *n* (%)	6 (3.2)	0 (0.0)
Dyslipidaemia, *n* (%)	150 (81.1)	110 (58.8)
Smokers, *n* (%)	11 (5.9)	20 (10.7)

Values are presented as mean (standard deviation) for continuous variables and as number (%) for categorical variables.

BSA, body surface area; SBP, systolic blood pressure; DBP, diastolic blood pressure.

### Image segmentation

All cine CMR sequences were segmented using a five-model ensemble of publicly available nnU-Net models for short- and long-axis cardiac CMR segmentation.^29,30^ The segmentation labels were used to extract anatomical contours for subsequent mesh fitting. Segmentation performance was evaluated at end-diastole in a subset of participants by comparing automated contours with expert manual delineations performed in Segment, version 4.1.0.1, Medviso, Lund, Sweden. Segmentation accuracy was quantified using Dice similarity coefficients for the left ventricular (LV) blood pool, right ventricular (RV) blood pool, and LV myocardium in short-axis views. Detailed validation results are reported in the Supplementary data.

### Biventricular mesh fitting

Cine acquisitions were corrected for breath-hold-related slice offsets before mesh reconstruction. The respiratory-gated three-dimensional thorax acquisition was used as a spatial reference and was resampled into the cine imaging planes. In-plane offsets in short-axis cine slices were estimated from the displacement between LV blood-pool centroids in the cine-derived and thorax-derived segmentations. For long-axis views, an additional through-plane correction was estimated by identifying the resampled thorax slice with the highest overlap with the corresponding cine segmentation. The thorax acquisition was used only for spatial alignment and not for mesh reconstruction. Full details of the correction procedure are provided in the Supplementary data.

A template biventricular mesh was aligned to each subject using an initial similarity transformation based on anatomical landmarks. Global scaling, rigid rotation, and translation were estimated using a Kabsch-based alignment procedure.^29,31^

After initial alignment, non-rigid mesh fitting was performed using a hierarchical free-form deformation framework.^32^ Coarse and fine deformation grids were optimized to minimize distances between image-derived contours and the corresponding mesh surfaces while enforcing smoothness and regularity of the deformation field. The fitted structures included the LV endocardium, LV epicardium, RV endocardium, septum, valve planes, and great-vessel regions. Because right ventricular myocardial labels were not available from the segmentation network, the RV endocardial surface was fitted directly, whereas the RV epicardial surface was constrained by deformation regularization to preserve biventricular topology. Consequently, right ventricular interpretations were restricted to chamber geometry and global biventricular form.

Mesh fitting was implemented in PyTorch and performed on GPU hardware, NVIDIA TITAN XP, 12 GB VRAM, using the Adam optimizer for 200 iterations per cardiac frame. The mesh fitting pipeline is publicly available at https://gitlab.ethz.ch/ibt-cmr-public/cmrpro.

Method verification included comparison of automated segmentations with expert manual contours and comparison of mesh-derived ventricular volumes with segmentation-derived volumes across the cohort. Detailed verification results are reported in the Supplementary data.

### Classical biomarker calculations

LV myocardial mass (LVM) was calculated from the fitted mesh assuming a myocardial density of 1.055 g/mL. LV wall thickness (LVWT) was computed as the mean distance between corresponding LV endocardial and epicardial points. LV and RV blood pool volumes were obtained by closing the endocardial surfaces and computing the enclosed volume. For both ventricles, end-diastolic volume (EDV) and end-systolic volume (ESV) were defined as the maximum and minimum chamber volumes over the cardiac cycle, respectively. EF was computed as $\mathrm{EF}=\frac{\mathrm{EDV}-\mathrm{ESV}}{\mathrm{EDV}}\times 100$. LV sphericity at end-diastole (ED) $\psi_{\mathrm{ED}}$ was computed as $\psi_{\mathrm{ED}}=\frac{V}{\frac{4}{3}\pi {\left(\frac{L}{2}\right)}^{3}}$ where *V* is the blood pool volume and Lis mitral valve (MV)-apex distance at ED.^33^ Classical biomarkers were compared with published reference ranges.^4-6^

### Statistical biventricular shape models

End-diastolic biventricular meshes were used for statistical shape modelling. Meshes were centred and rigidly aligned to establish a common coordinate frame while preserving global anatomical scale. Scale was retained so that the resulting shape models represented anatomical form, including both size- and shape-related variation. Alignment was performed by orienting the apex-to-mitral-valve axis with the z-axis and the left-ventricular-to-right-ventricular direction with the negative x-axis.

For each participant, the coordinates of all mesh vertices were concatenated into a single shape vector. Principal component analysis was then applied to derive orthonormal modes of biventricular anatomical variation. Modes explaining 95% of cumulative variance were retained. Principal component analysis (PCA) was used because it provides a linear, compact, and directly visualizable representation of population-level shape variation.^15,16^ Three PCA models were constructed: one pooled sex-agnostic model using the full cohort, one male-specific model, and one female-specific model. Pooled model modes are denoted PC_G_, male-specific modes PC_M_, and female-specific modes PC_F_. Scores associated with the *i*-th PCA mode are denoted by z*^i^*, with subscript G, M, F indicating the sex-agnostic, male-specific and female-specific models, respectively.

Mode correspondence between PCA spaces was assessed using the absolute scalar product between normalized PCA mode vectors represented in the common vertex-coordinate space. Values close to 0 indicate near-orthogonal anatomical directions, whereas values close to 1 indicate near-identical directions of anatomical variation. The absolute value was used because the sign of PCA modes is arbitrary.

For each mode of the pooled sex-agnostic model, score variance was decomposed into male within-sex variance, female within-sex variance, and between-sex variance. Within-sex components were computed from deviations of individual scores from the corresponding sex-specific mean score. The between-sex component was computed from the weighted squared deviation of each sex-specific mean score from the overall cohort mean score. Each component was expressed as a percentage of total score variance for that mode. This analysis was used to determine whether pooled PCA modes primarily reflected within-sex anatomical variation or differences in mean anatomy between sexes.

### Statistical analysis

Continuous variables are reported as mean and standard deviation, and categorical variables as counts and percentages. Age-stratified summaries were used for descriptive visualization only. Primary analyses treated age as a continuous variable.

Associations between anatomical PCA scores and conventional CMR biomarkers were assessed using Pearson correlation coefficients. These correlations were used to aid anatomical interpretation of PCA modes and were not treated as primary inferential tests. Multicollinearity among regression covariates was assessed using Pearson correlation coefficients and variance inflation factors.

The primary analysis tested associations between anatomical PCA scores and age. For each retained PCA mode, the PCA score was modelled as the dependent variable and age as the predictor of interest. Models were adjusted for body surface area, heart rate, systolic blood pressure, and diastolic blood pressure. Sex-specific PCA spaces were analysed separately in males and females. The pooled sex-agnostic PCA space was analysed using a combined model including sex, age, and an age-by-sex interaction, with females coded as 0 and males coded as 1.

Models were fitted using ordinary least squares regression with HC3 heteroskedasticity-robust standard errors. Regression coefficients are reported with robust 95% confidence intervals. Multiple comparisons were controlled using the Benjamini–Hochberg false discovery rate across all retained PCA modes within each model group. For the pooled sex-agnostic model, correction was applied separately to the age effect and the age-by-sex interaction. Unless otherwise specified, reported *P-*values are -corrected for false discovery rate (FDR), whereas confidence intervals are unadjusted robust confidence intervals.

The incremental contribution of age was quantified by comparing each full model with a covariate-only reduced model. The increase in explained variance is reported as $\Delta R^{2}$, and Cohen’s *f*^2^ was computed from the change in explained variance.

To assess whether sex-specific shape spaces provided a more compact representation of clinically relevant anatomical variation, exploratory downstream prediction analyses were performed. Age prediction was evaluated using regression models including clinical covariates alone, or clinical covariates plus increasing numbers of leading PCA scores. Clinical covariates comprised body surface area, heart rate, systolic blood pressure, and diastolic blood pressure. Additional analyses assessed whether PCA scores improved prediction of conventional CMR biomarkers beyond age and clinical covariates. The biomarker endpoints were left ventricular end-diastolic volume (LVEDV), LVM, right ventricular end-diastolic volume (RVEDV), and LV end-diastolic sphericity.

Sex-specific and sex-agnostic shape representations were compared using the same number of leading PCA scores.

Downstream prediction models were evaluated using cross-validation within the screened cohort. For groups with at least 12 participants, five-fold cross-validation with shuffling was used. For smaller groups, leave-one-out cross-validation was used. Ridge regression models used internal selection of the regularization parameter from 25 logarithmically spaced alpha values. Model performance was summarized using root mean square error and *R*^2^.

The PCA shape representations were computed once before downstream prediction and were not recomputed within each cross-validation fold. These analyses should therefore be interpreted as exploratory comparisons of the clinical information contained in fixed sex-specific and sex-agnostic shape representations, rather than as fully nested end-to-end out-of-sample validation of the complete shape-modelling pipeline.

## Results

### Cohort and conventional biomarker analysis

Of the 382 screened participants eligible for this analysis, 10 were excluded because of automated segmentation or mesh-fitting failure, corresponding to a pipeline success rate of 97.4%. The final analysis cohort comprised 372 participants, including 185 males and 187 females.

Automated short-axis segmentations showed Dice similarity coefficients of 0.95 ± 0.03 for the LV blood pool, 0.90 ± 0.04 for the right ventricular blood pool, and 0.82 ± 0.05 for the LV myocardium. Mesh-derived ventricular volumes showed close agreement with segmentation-derived volumes. Additional verification results are reported in the Supplementary data.

Sex- and age-stratified conventional CMR biomarkers are summarized in *Tables 2* and *3* (for the full SwissHeart cohort, see *Tables A2* and *A3* in the Supplementary data). Females had smaller ventricular volumes and higher EF than males. Ventricular volumes were lower in older age groups in both sexes, particularly in females. *Figure 2* shows age-stratified mean end-diastolic anatomical differences relative to the 18–29-year reference group. The oldest age group should be interpreted cautiously because of the small number of participants.

**Figure 2:**
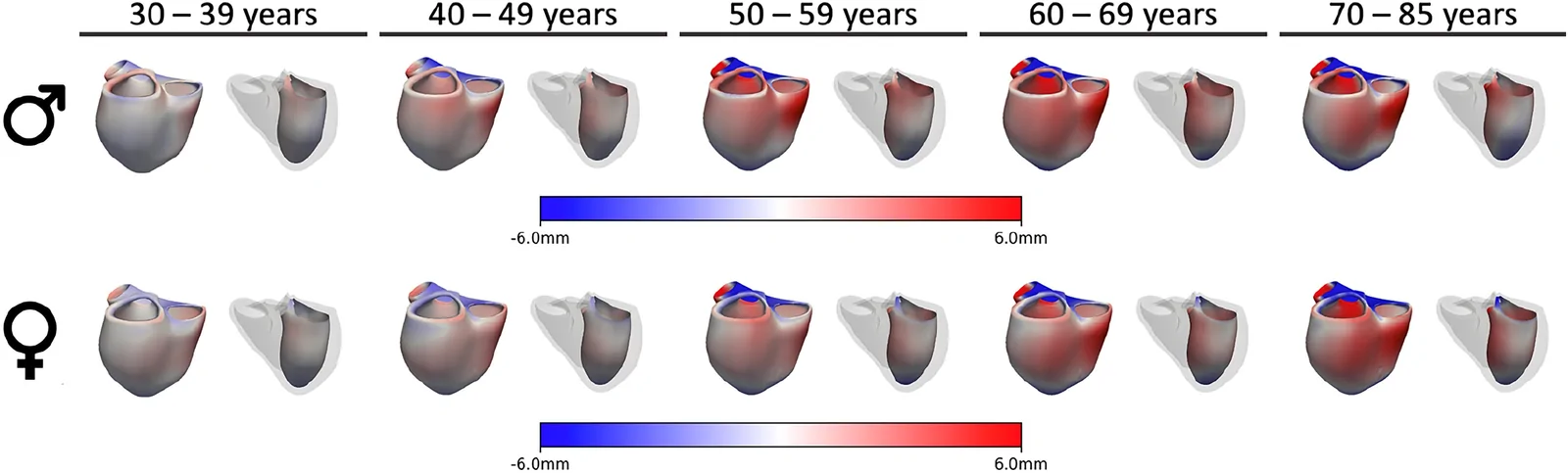
Age-associated differences in end-diastolic ventricular anatomy in males (top row) and females (bottom row), shown as decade-wise group averages relative to the youngest cohort (18–29 years). The colour field represents the signed radial difference between each age-group mean anatomy and the reference anatomy. Positive values indicate an outward spatial offset relative to the reference surface, along the local endocardium-to-epicardium direction, whereas negative values indicate the opposite direction. These values describe between-group anatomical differences and do not represent myocardial motion.

**Table 2:** Age-stratified cardiac metrics for the male cohort included in this analysis

Biomarker		Age groups					
		18–29	30–39	40–49	50–59	60–69	70–85
*n*	—	45	47	35	33	19	6
Weight	kg	78 (11)	81 (13)	81 (11)	85 (14)	80 (11)	84 (12)
Height	cm	180 (7)	182 (8)	182 (7)	181 (7)	180 (5)	178 (9)
BSA	m^2^	1.96 (0.16)	2.02 (0.19)	2.02 (0.15)	2.06 (0.19)	2.00 (0.15)	2.04 (0.20)
SBP	mmHg	127 (11)	127 (12)	128 (13)	135 (14)	135 (17)	148 (6)
DBP	mmHg	74 (8)	76 (8)	78 (11)	84 (8)	82 (10)	81 (8)
LVEDV	mL	150 (20)	153 (24)	142 (23)	140 (23)	133 (24)	141 (31)
LVESV	mL	61 (12)	61 (12)	55 (11)	56 (14)	53 (13)	53 (11)
LVEF	%	59 (5)	60 (4)	61 (4)	60 (5)	60 (5)	62 (4)
LVM	g	124 (18)	127 (21)	126 (21)	125 (23)	118 (24)	129 (18)
LVWT	mm	6.6 (0.5)	6.7 (0.5)	6.8 (0.5)	6.7 (0.6)	6.6 (0.5)	6.8 (0.3)
RVEDV	mL	150 (24)	152 (25)	141 (25)	142 (26)	130 (25)	139 (31)
RVESV	mL	62 (15)	62 (15)	56 (16)	57 (15)	55 (13)	53 (11)
RVEF	%	59 (6)	60 (5)	61 (6)	60 (6)	58 (6)	61 (3)
$\Psi_{ED}$	—	0.25 (0.04)	0.25 (0.04)	0.23 (0.04)	0.25 (0.04)	0.25 (0.05)	0.27 (0.05)

Values are presented as mean (standard deviation) for weight, height, BSA, systolic and diastolic blood pressures (SBP, DBP), left and right ventricular end-diastolic and end-systolic volumes (LVEDV, LVESV, RVEDV, RVESV), ejection fraction (LVEF, RVEF), LV myocardium mass (LVM), LV wall thickness (LVWT), sphericity at end-diastole (*Ψ*_ED_).

BSA, body surface area; SBP, systolic blood pressure; DBP, diastolic blood pressure; LVEDV, left ventricular end-diastolic volume; LVESV, left ventricular end-systolic volume; RVEDV, right ventricular end-diastolic volume; RVESV, right ventricular end-systolic volume; LVEF, left ventricular ejection fraction; RVEF, right ventricular ejection fraction; LVM, left ventricular myocardial mass; LVWT, left ventricular wall thickness.

**Table 3:** Age-stratified cardiac metrics for the female cohort included in this analysis

Biomarker		Age groups					
		18–29	30–39	40–49	50–59	60–69	70–85
*n*	—	40	40	41	37	23	6
Weight	kg	64 (8)	63 (7)	64 (8)	65 (8)	64 (10)	60 (8)
Height	cm	169 (6)	168 (7)	167 (5)	166 (5)	165 (6)	162 (9)
BSA	m^2^	1.72 (0.13)	1.71 (0.12)	1.72 (0.12)	1.73 (0.11)	1.71 (0.13)	1.64 (0.15)
SBP	mmHg	116 (10)	115 (11)	114 (12)	119 (11)	129 (16)	137 (20)
DBP	mmHg	76 (7)	76 (10)	76 (8)	79 (7)	82 (11)	78 (13)
LVEDV	mL	115 (17)	111 (17)	103 (15)	98 (10)	93 (13)	90 (19)
LVESV	mL	44 (7)	41 (7)	37 (8)	35 (5)	33 (6)	29 (8)
LVEF	%	62 (3)	63 (4)	64 (4)	65 (4)	64 (5)	68 (3)
LVM	g	91 (14)	86 (15)	80 (16)	79 (9)	81 (10)	74 (11)
LVWT	mm	5.9 (0.4)	5.8 (0.4)	5.7 (0.5)	5.7 (0.4)	5.8 (0.5)	5.8 (0.3)
RVEDV	mL	112 (18)	106 (18)	95 (16)	94 (12)	88 (17)	80 (18)
RVESV	mL	44 (11)	38 (10)	33 (9)	32 (6)	31 (8)	27 (10)
RVEF	%	61 (5)	64 (5)	66 (5)	66 (4)	65 (6)	67 (6)
$\Psi_{ED}$	—	0.23 (0.05)	0.25 (0.04)	0.24 (0.04)	0.25 (0.05)	0.25 (0.06)	0.26 (0.05)

Values are presented as mean (standard deviation) for weight, height, body surface area (BSA), systolic and diastolic blood pressures (SBP, DBP), left and right ventricular end-diastolic and end-systolic volumes (LVEDV, LVESV, RVEDV, RVESV), ejection fraction (LVEF, RVEF), LV myocardial mass (LVM), LV wall thickness (LVWT), sphericity at end-diastole (*Ψ*_ED_).

### Variance structure and between-sex contribution of PCA models

The sex-agnostic PCA model retained 17 modes to explain 95% of cumulative shape variance. The male-specific model retained 17 modes, and the female-specific model retained 18 modes.

The first mode of the sex-agnostic model explained 31% of total shape variance, compared with 22% and 19% for the first male- and female-specific modes, respectively (*Figure 3*). From the second mode onwards, the proportion of variance explained by individual modes was more similar across models.

**Figure 3:**
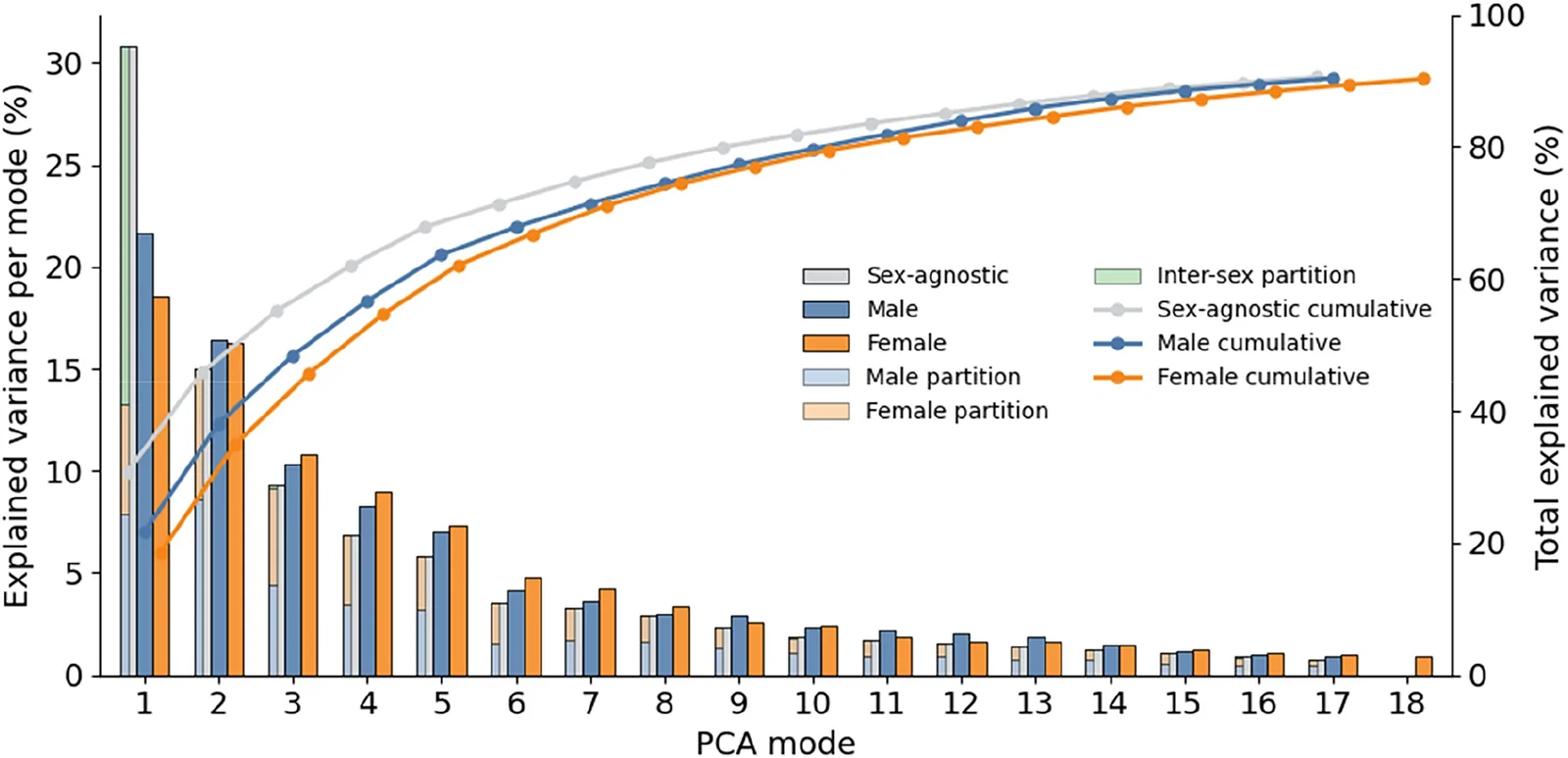
Explained variance and sex-wise partitioning of sex-agnostic PCA modes. Bars show the percentage of variance explained by each PCA mode for the sex-agnostic, male-specific, and female-specific shape models. Lines show the corresponding cumulative explained variance. Stacked bars indicate the decomposition of sex-agnostic score variance into male within-sex, female within-sex, and between-sex components for each mode.

Variance decomposition showed that the first sex-agnostic mode contained a substantial between-sex component. Between-sex anatomical differences accounted for 57% of its variance, whereas within-male and within-female variance accounted for 26% and 17%, respectively (*Figure 3*). Subsequent sex-agnostic modes showed minimal between-sex contribution, below 1%, with most variance attributable to within-sex variation.

Cosine similarity analysis showed that correspondence between sex-agnostic and sex-specific PCA modes was not strictly index-preserving (see *Figure A1*). Leading modes showed partial alignment across model spaces, whereas higher-order modes showed more dispersed correspondence patterns.

### Anatomical interpretation of leading modes

*Figure 4* shows anatomical variation induced by ±4 SD perturbations along the first five modes of each model. Quantitative changes in conventional anatomical biomarkers are reported in *Figure A2*.

**Figure 4:**
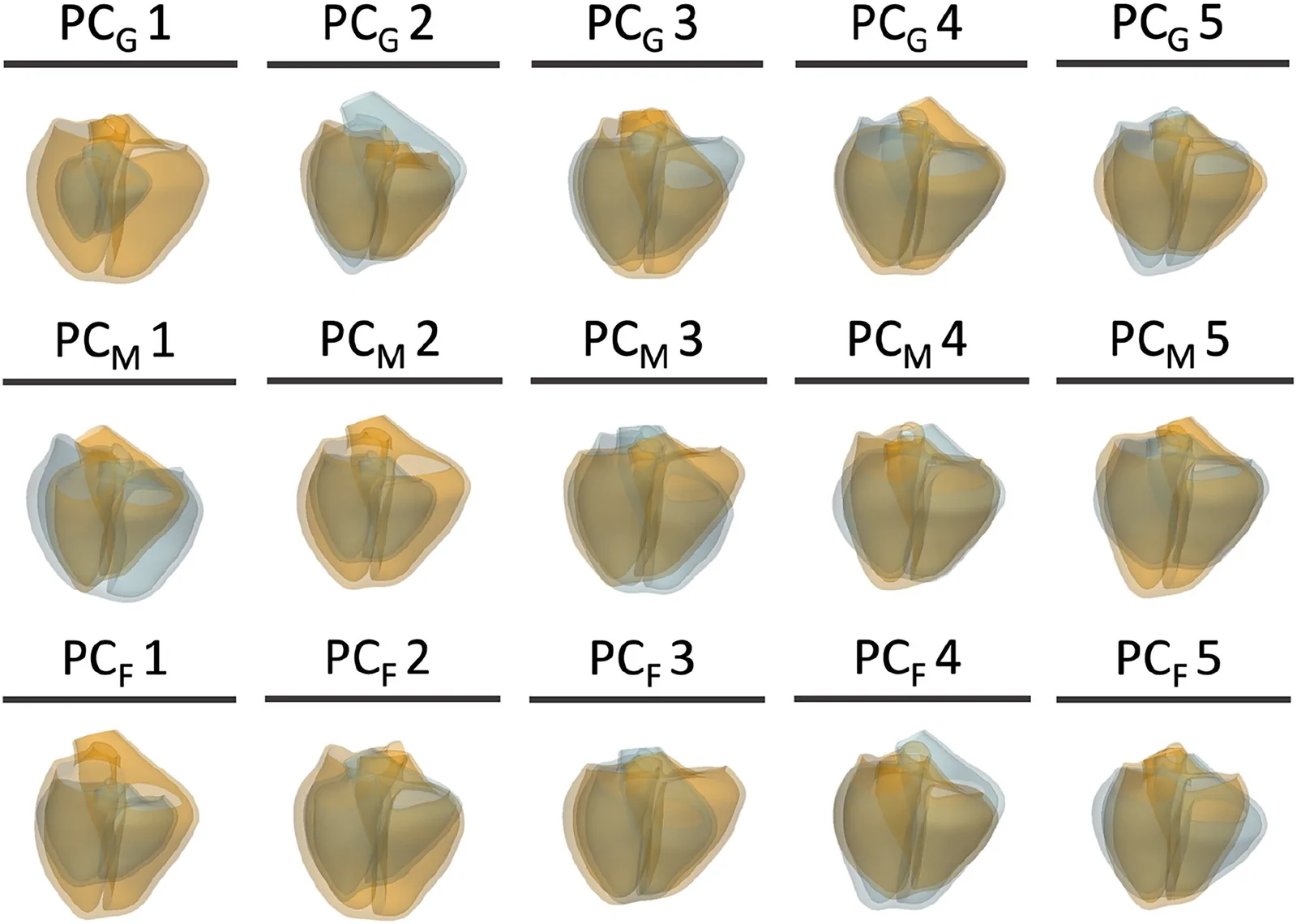
Top five leading anatomical modes for the shape models. Top row: sex-agnostic (PC_G_); middle row: male sex-specific (PC_M_); bottom row: female-specific model (PC_F_). Shape variations are shown for ±4 SDs of each anatomical score, with negative scores in light orange and positive scores in light blue.

In the sex-agnostic model, the first mode represented global cardiac size variation, with coordinated changes in left and right ventricular chamber volumes and LVM. The second global mode was associated with variation in LV sphericity and wall-thickness measures, with smaller changes in ventricular volumes. The third mode encoded sphericity-related changes in the anatomy, accompanied by tilting of mitral and tricuspid valves (MV and TV, respectively) planes and a variation of the LV/RV volume ratio. The fourth global mode represented minor changes in variation of sphericity, and a variation in the distance between the LV–RV junctions. The fifth global mode mainly reflected sphericity-related variation affecting both ventricles.

In males, the first sex-specific mode included changes in ventricular size and myocardial mass, with large sphericity-related changes. The second mode showed larger changes in ventricular volumes and wall-thickness-related measures, with limited change in sphericity, leading to mostly global scaling. The third and fifth modes determined large changes in biventricular sphericity as well, while mode four impacted predominantly LV sphericity and a variation in the volume ratio between LV and RV.

In females, the first mode combined changes in sphericity with reductions in blood-pool volume, myocardial mass, and wall-thickness-related measures. The second female-specific mode showed a non-symmetric impact on LV and RV, with increased RV sphericity and, with respect to LV, a variation in the volume ratio between the ventricles. The third mode combined volume- and sphericity-related variation, equally distributed on both ventricles, with a reorientation of valve planes. The fourth female-specific mode determined minor changes in the anatomy of the apical and basal regions, while the fifth mode determined almost uniquely large variations in biventricular sphericity.

### Association between anatomical scores and clinical biomarkers

*Figure 5* summarizes Pearson correlations between anatomical PCA scores and conventional CMR biomarkers.

**Figure 5:**
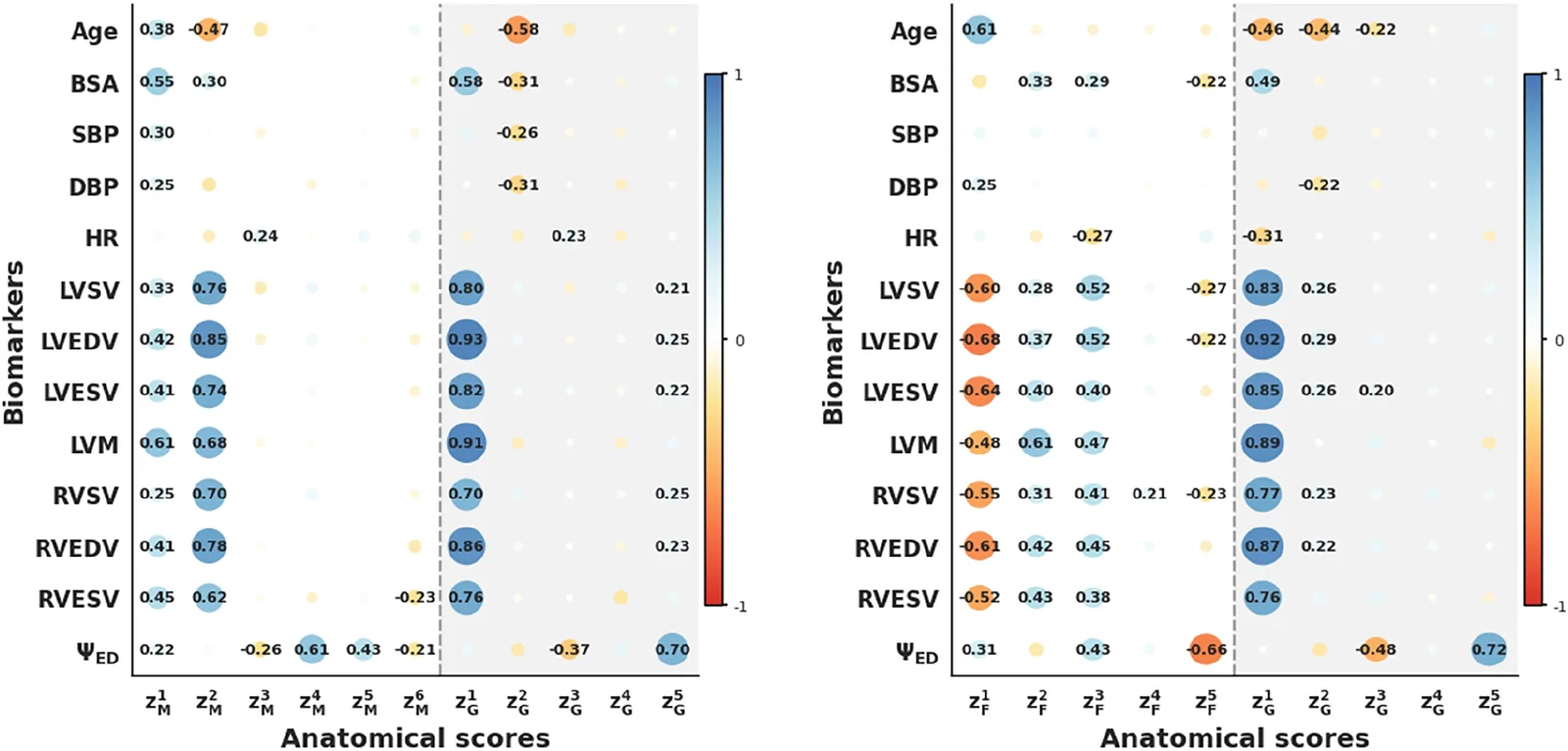
Correlations between anatomical scores and clinical biomarkers in sex-specific and sex-agnostic models. Bubble plots show Pearson correlations between anatomical PCA scores and clinical biomarkers for males, left and females, right. Within each panel, scores from the sex-specific model are shown on the left of the dashed vertical line, and scores from the sex-agnostic model are shown on the right. Bubble size indicates correlation magnitude, and colour indicates correlation direction and strength. Positive correlations are shown in blue and negative correlations in red. Only correlation coefficients with absolute magnitude above 0.2 are labelled.

In males, the strongest volume-related correlations in the sex-specific model were observed for PC_M_2, which correlated with left and right ventricular volume and mass biomarkers, with correlation coefficients ranging from 0.62 to 0.85. PC_M_1 also correlated positively with body surface area, ventricular volumes, and LVM, but with lower coefficients.

In females, PC_F_1 showed a positive association with age and negative correlations with ventricular volumes and LVM, with correlation coefficients ranging from −0.48 to −0.68 for volume and mass biomarkers. PC_F_2 and PC_F_3 showed positive correlations with body surface area, ventricular volumes, and LVM.

In the sex-agnostic model, PC_G_1 showed strong positive correlations with body surface area, ventricular volumes, and LVM in both sexes. PC_G_2 showed stronger associations with age-associated variation and was inversely associated with age in both males and females. Sphericity-related variation was represented by higher-order global modes.

### Covariate-adjusted age associations in sex-specific and sex-agnostic models

Variance inflation factors were below 3 for all regression covariates, indicating no substantial multicollinearity among age, body surface area, heart rate, systolic blood pressure, and diastolic blood pressure. Scatter plots of age against the leading PCA scores are shown in *Figure A3*.

In the male-specific PCA space, age was significantly associated with PC_M_1, PC_M_2, and PC_M_6 after false-discovery-rate correction (*Table 4*). The strongest association was observed for PC_M_2, with *β* = 14.8 × 10^−3^ PCA score units per year, 95% CI 11.9 to 17.7, *P* < 0.001, Δ*R*^2^ = 0.251, and Cohen’s *f*^2^ = 0.451. PC_M_1 showed a smaller but significant association with age, *β* = 9.4 × 10^−3^ PCA score units per year, 95% CI 5.7 to 13.1, *P* < 0.001, Δ*R*^2^ = 0.076, and *f*^2^ = 0.133. PC_M_6 showed a weaker association, *β* = −2.5 × 10^−3^ PCA score units per year, 95% CI −4.4 to −0.6, *P* = 0.038, Δ*R*^2^ = 0.028, and *f*^2^ = 0.030.

**Table 4:** Multivariable associations between age and sex-specific anatomical PCA scores

Males					Females				
Mode	$\beta \times 10^{-3}$	*P*	Δ*R*^2^	*f* ^2^	Mode	$\beta \times 10^{-3}$	*P*	Δ*R*^2^	*f* ^2^
PC_M_1	9.4 (5.7, 13.1)	<0.001	0.076	0.133	PC_F_1	15.6 (12.8, 18.4)	<0.001	0.335	0.619
PC_M_2	14.8 (11.9, 17.7)	<0.001	0.251	0.451	PC_F_2	2.4 (−1.5, 6.4)	0.318	0.009	0.011
PC_M_3	−3.4 (−6.7, −0.0)	0.138	0.021	0.023	PC_F_3	2.5 (−0.4, 5.5)	0.173	0.015	0.019
PC_M_4	1.7 (−1.2, 4.6)	0.330	0.006	0.006	PC_F_4	−1.4 (−4.2, 1.4)	0.381	0.006	0.006
PC_M_5	0.6 (−2.2, 3.4)	0.735	0.001	0.001	PC_F_5	2.0 (−0.4, 4.3)	0.188	0.013	0.015
PC_M_6	−2.5 (−4.4, −0.6)	0.038	0.028	0.030	PC_F_6	−1.6 (−3.8, 0.5)	0.198	0.014	0.015
PC_M_7	0.0 (−2.2, 2.2)	0.994	0.000	0.000	PC_F_7	1.8 (−0.2, 3.9)	0.173	0.020	0.021

Age effects are reported as regression coefficients, *β*, in PCA score units per year, with HC3 robust 95% confidence intervals. *P-*values are FDR-corrected across all retained modes within each sex-specific PCA model. Δ*R*^2^ denotes the incremental variance explained by adding age to the covariate-only model, and *f*^2^ denotes Cohen’s effect size. *β* values are scaled by 10^−3^ PCA score units per year. Only the first seven modes are shown.

In the female-specific PCA space, age-associated variation was concentrated in PC_F_1. Age was significantly associated with PC_F_1 after false-discovery-rate correction, with *β* = 15.6 × 10^−3^ PCA score units per year, 95% CI 12.8 to 18.4, *P* < 0.001, Δ*R*^2^ = 0.335, and *f*^2^ = 0.619. No other female-specific mode remained significantly associated with age after correction.

In the sex-agnostic PCA space, PC_G_2 showed the largest incremental contribution of age and the age-by-sex interaction terms, with Δ*R*^2^ = 0.220 and *f*^2^ = 0.324 (*Table 5*). Age was significantly associated with PC_G_2 in both females and males, with negative slopes in both sexes. The age-by-sex interaction for PC_G_2 did not remain significant after correction, *P* = 0.086.

**Table 5:** Multivariable associations between age, sex, and sex-agnostic anatomical PCA scores

Mode	$\beta_{F}\times 10^{-3}$	*P* Female	$\beta_{M}\times 10^{-3}$	*P* Male	$\beta_{M}-{\beta^{\circ }}_{F}\times 10^{-3}$	*P*	Δ*R*^2^	*f* ^2^
PC_G_1	10.6 (8.3, 12.9)	<0.001	5.4 (2.4, 8.4)	0.002	−5.2 (−8.9, −1.5)	0.044	0.045	0.209
PC_G_2	−10.7 (−13.8, −7.5)	<0.001	−15.7 (−19.1, −12.3)	<0.001	−5.0 (−9.4, −0.6)	0.086	0.220	0.324
PC_G_3	4.5 (1.1, 7.9)	0.023	2.9 (−0.2, 6.0)	0.094	−1.6 (−6.3, 3.0)	0.579	0.030	0.032
PC_G_4	0.2 (−2.7, 3.0)	0.909	0.0 (−2.7, 2.7)	0.980	−0.1 (−4.0, 3.7)	0.948	0.000	0.000
PC_G_5	−2.3 (−4.6, −0.0)	0.083	0.7 (−1.9, 3.2)	0.612	3.0 (−0.4, 6.3)	0.186	0.011	0.011
PC_G_6	−1.3 (−3.3, 0.8)	0.323	−3.0 (−5.4, −0.6)	0.036	−1.7 (−4.7, 1.2)	0.367	0.027	0.028
PC_G_7	−0.6 (−2.3, 1.1)	0.593	−2.0 (−4.0, −0.1)	0.068	−1.4 (−4.0, 1.1)	0.257	0.012	0.013

Age effects are reported as regression coefficients, *β*, in $10^{-3}$ score units per year, with HC3 robust 95% confidence intervals. $\beta_{F}$ denotes the female age slope, $\beta_{M}$ denotes the male age slope. *P* Female, *P* Male, and *P* represent the FDR-corrected values for females, males, and the age-by-sex interaction. $\Delta R^{2}$ denotes the incremental variance explained by adding age and the age-by-sex interaction to the covariate-only model, and $f^{2}$ denotes Cohen’s effect size. Only the first seven modes are presented.

PC_G_1 also showed significant age associations in both females and males, with positive age slopes in both sexes. The age-by-sex interaction for PC_G_1 remained significant after correction, *P* = 0.044. Smaller age associations were observed for PC_G_3 in females and PC_G_6 in males. No other age-by-sex interaction remained significant after correction.

### Exploratory downstream prediction analyses

Exploratory downstream prediction analyses compared sex-specific and sex-agnostic shape representations using increasing numbers of leading PCA scores (*Figure 6*). With only the leading PCA mode retained, sex-specific models showed lower age-prediction error than the sex-agnostic model in both sexes, with root mean squared error (RMSE) values of 12.6 vs. 13.5 years in males and 11.3 vs. 12.1 years in females.

**Figure 6:**
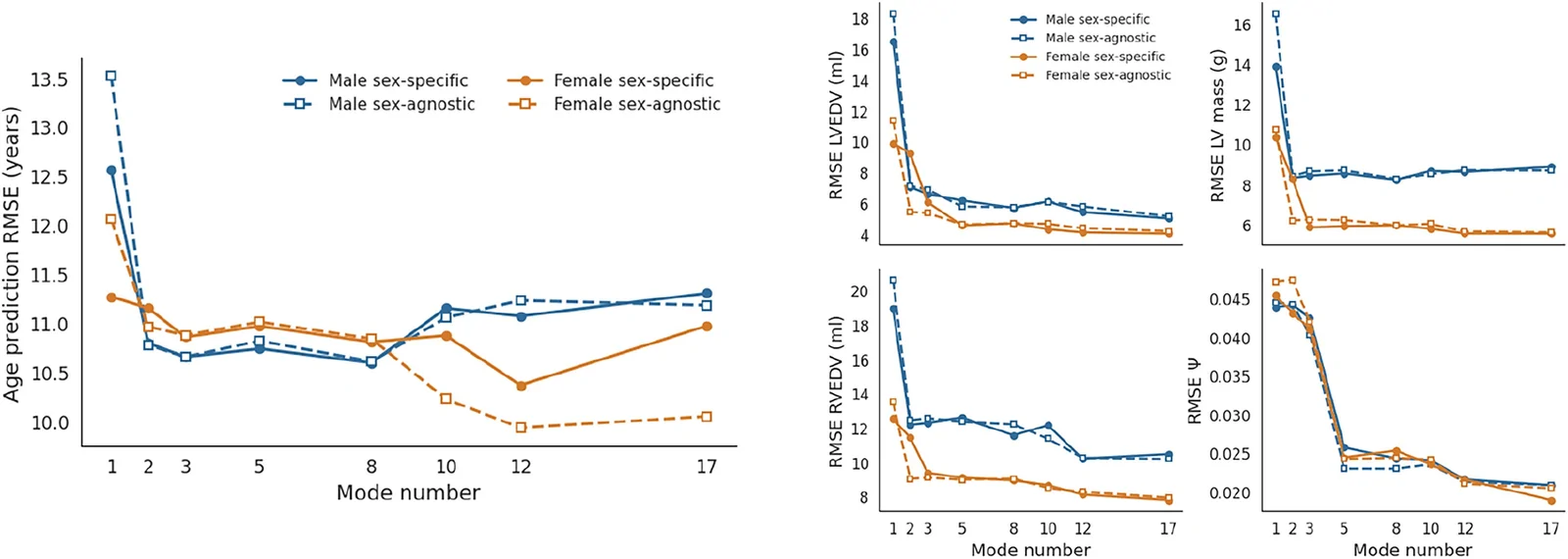
Comparison of sex-specific and sex-agnostic anatomical statistical shape models across an increasing number of retained PCA modes. Left: cross-validated age prediction error for sex-specific and sex-agnostic models expressed as RMSE. Right: biomarker prediction error for sex-specific and sex-agnostic models expressed as RMSE for LVEDV, LVM, RVEDV, and end-diastolic sphericity *Ψ*.

A similar single-mode pattern was observed for ventricular volume and mass biomarkers. Sex-specific models showed lower RMSE for LVEDV, RVEDV, and LVM in both sexes, whereas differences for end-diastolic sphericity were small. With additional retained modes, performance differences between sex-specific and sex-agnostic representations became smaller and less consistent across endpoints.

## Discussion

In this cross-sectional CMR study, sex-agnostic and sex-specific statistical shape models provided complementary but non-equivalent representations of end-diastolic biventricular anatomy. The main finding was that the pooled model was dominated by between-sex anatomical variation, whereas sex-specific models organized within-sex age-associated anatomical variation into more prominent modes. This was supported by the variance decomposition in *Figure 3*, anatomical mode visualizations in *Figure 4*, biomarker correlations in *Figure 5*, and adjusted age-association analyses in *Tables 4* and *5*.

The first pooled mode explained 31% of total shape variance and contained a large between-sex component, 57% of mode variance, compared with 26% within males and 17% within females (*Figure 3*). This mode was also strongly associated with body surface area, ventricular volumes, and myocardial mass (*Figure 5*). It therefore described total population variability well, but was less specific for within-sex age-associated anatomy.

Sex-specific models provided a more direct representation of age-associated variation within each sex. In males, the strongest adjusted age association was observed for PC_M_2, which also correlated strongly with ventricular volume and mass biomarkers (*Figure 5* and *Table 4*). In females, age-associated variation was concentrated in PC_F_1, which was inversely associated with ventricular volumes and myocardial mass (*Figure 5* and *Table 4*). These findings are consistent with the descriptive biomarker summaries in *Tables 2* and *3* and with the anatomical perturbations shown in *Figure 4* and Figure A2.

The pooled model also captured age-associated variation, but this information was distributed across multiple modes. PC_G_2 showed the largest incremental contribution of age and age-by-sex interaction terms, while PC_G_1 also showed significant age associations in both sexes (*Table 5*). Because PC_G_1 was strongly influenced by between-sex anatomy, age-associated information in the pooled model was partly mixed with broader cohort structure. The cosine similarity analysis in Figure A1 further showed that anatomical correspondence between pooled and sex-specific modes was not strictly index-preserving.

The exploratory prediction analyses in *Figure 6* supported this interpretation. With only one retained mode, sex-specific models showed lower age-prediction error and generally lower biomarker-prediction error than the sex-agnostic model. With additional modes, performance differences became smaller and less consistent. Thus, the main value of sex-specific modelling was not improved overall prediction, but a more compact and anatomically interpretable organization of within-sex variation.

Together, these findings support the use of sex-specific statistical shape models as complementary reference spaces for population CMR phenotyping. Pooled models remain useful for describing total anatomical variability, whereas sex-specific models may be preferable when the objective is to characterize interpretable within-sex patterns of age-associated ventricular anatomy.

From a clinical translation perspective, the automated segmentation and mesh-fitting pipeline succeeded in 97.4% of eligible cases, supporting technical feasibility for cohort-scale research, and it requires standard CMR acquisitions. The present implementation requires quality control of segmentations and meshes, but future streamlined quality-control procedures could improve deployability in a clinical setting. Once a validated reference space is established, sex-specific scores could provide standardized and anatomically interpretable early measures of an individual's deviation from the reference population. Potential clinical applications would be the assessment of patients with suspected pathological progression whose ventricular volumes, myocardial mass, and EF remain within conventional reference ranges. A pooled model may be less sensitive to this deviation because its leading modes also encode normal differences between male and female anatomy. Such low-dimensional anatomical scores could also provide interpretable input features for machine-learning models, potentially retaining coordinated three-dimensional anatomical information that is not represented by conventional scalar biomarkers.

Before clinical implementation, processing time, test-retest reproducibility, robustness across scanners and acquisition protocols, prognostic value, and incremental clinical utility beyond conventional CMR biomarkers will require prospective evaluation.

## Limitations

This study has several limitations. First, the cross-sectional design does not allow direct inference of longitudinal ventricular remodelling. The reported age effects represent population-level age associations rather than within-subject anatomical trajectories, and may reflect cohort effects, residual cardiovascular risk, lifestyle factors, or unmeasured confounders, as previously shown in MESA.^28^

Secondly, the cohort was restricted to screened Caucasian/White Swiss adults without manifest cardiovascular disease, which limits generalizability to other ethnic groups and populations with different cardiovascular risk profiles. Nevertheless, Moscoloni *et al*. reported broadly consistent sex-associated patterns in a healthy UK Biobank cohort, including major contributions from chamber size and relative ventricular positioning and persistent sex-related morphological differences after adjustment for age, blood pressure, and body size.^21^ This agreement across two European cohorts supports the robustness of the principal finding that sex is an important determinant of biventricular anatomy, but it does not establish the transportability of the present PCA basis, score distributions, or age-associated patterns. External validation in ethnically and geographically diverse cohorts, using harmonized imaging and modelling procedures, is required before these models can serve as clinical reference standards. Although overt cardiovascular disease and hypertension were excluded, residual risk factors such as dyslipidaemia, smoking, diabetes, or elevated measured blood pressure may still have influenced anatomical variation.

Thirdly, the analysis relied on automated segmentation and mesh fitting. Although method verification showed good segmentation accuracy and close agreement between mesh-derived and segmentation-derived volumes, residual errors may affect localized anatomical features. Because right ventricular myocardial labels were not available, right ventricular interpretations should be limited to chamber geometry and global biventricular form.

Finally, PCA provides a linear and sample-dependent representation of anatomical variability.

## Conclusions

Sex-specific statistical shape models provided a complementary representation of end-diastolic biventricular anatomy compared with a pooled sex-agnostic model. In this cross-sectional CMR cohort, the pooled model encoded sex as the dominant covariate direction, whereas sex-specific models concentrated within-sex age-associated anatomical variation into leading modes. These findings support the use of sex-specific anatomical reference spaces when the objective is to characterize interpretable patterns of ventricular shape variation within sex.

## Supplementary Material

qyag156_Supplementary_Data

## Data Availability

The source code for mesh fitting is publicly available at https://gitlab.ethz.ch/ibt-cmr-public/cmrpro. Access to the data may be considered on reasonable request, subject to the applicable ethics and data-sharing agreement.

## Lead author biography

Stefano Buoso is a senior scientist in biomedical engineering with expertise in cardiovascular magnetic resonance imaging, computational cardiac modelling, and data-driven analysis of heart anatomy and function. His work focuses on using advanced imaging, statistical shape modelling, and machine learning to better understand cardiovascular structure, ageing, and disease. His research bridges engineering, medical imaging, and clinical science, with the aim of developing robust, interpretable tools for biomedical research and translational cardiovascular medicine.

## References

[1] Cikes M, Solomon SD. Beyond ejection fraction: an integrative approach for assessment of cardiac structure and function in heart failure. Eur Heart J 2016;37:1642–50. 10.1093/eurheartj/ehv510 26417058

[2] Scatteia A, Baritussio A, Bucciarelli-Ducci C. Strain imaging using cardiac magnetic resonance. Heart Fail Rev 2017;22:465–76. 10.1007/s10741-017-9621-8 PMC5487809 28620745

[3] Xue H, Artico J, Davies RH, Adam R, Shetye A, Augusto JB et al Automated in-line artificial intelligence measured global longitudinal shortening and mitral annular plane systolic excursion: reproducibility and prognostic significance. J Am Heart Assoc 2022;11:e023849. 10.1161/JAHA.121.023849 PMC9245823 35132872

[4] Petersen SE, Aung N, Sanghvi MM, Zemrak F, Fung K, Paiva JM et al Reference ranges for cardiac structure and function using cardiovascular magnetic resonance (CMR) in Caucasians from the UK Biobank population cohort. J Cardiovasc Magn Reson 2016;19:18. 10.1186/s12968-017-0327-9 PMC5304550 28178995

[5] Kawel-Boehm N, Hetzel SJ, Ambale-Venkatesh B, Captur G, Francois CJ, Jerosch-Herold M et al Reference ranges (“normal values”) for cardiovascular magnetic resonance (CMR) in adults and children: 2020 update. J Cardiovasc Magn Reson 2020;22:87. 10.1186/s12968-020-00683-3 PMC7734766 33308262

[6] Raisi-Estabragh Z, Szabo L, McCracken C, Bulow R, Aquaro GD, Andre F et al Cardiovascular magnetic resonance reference ranges from the healthy hearts consortium. JACC Cardiovasc Imaging 2024;17:746–62. 10.1016/j.jcmg.2024.01.009 38613554

[7] Burch GE, Ray CT, Cronvich JA. The George Fahr Lecture: Certain mechanical peculiarities of the human cardiac pump in normal and diseased states. Circulation 1952;5:504–13. 10.1161/01.cir.5.4.504 14916466

[8] Burton AC . The importance of the shape and size of the heart. Am Heart J 1957;54:801–10. 10.1016/0002-8703(57)90186-2 13478518

[9] Gould KL, Lipscomb K, Hamilton GW, Kennedy JW. Relation of left ventricular shape, function and wall stress in man. Am J Cardiol 1974;34:627–34. 10.1016/0002-9149(74)90149-0 4279028

[10] Wong SP, French JK, Lydon AM, Manda SOM, Gao W, Ashton NG et al Relation of left ventricular sphericity to 10-year survival after acute myocardial infarction. Am J Cardiol 2004;94:1270–5. 10.1016/j.amjcard.2004.07.110 15541243

[11] Bai W, Shi W, de Marvao A, Dawes TJW, O'Regan DP, Cook SA et al A bi-ventricular cardiac atlas built from 1000+ high resolution MR images of healthy subjects and an analysis of shape and motion. Med Image Anal 2015;26:133–45. 10.1016/j.media.2015.08.009 26387054

[12] Bluemke DA, Kronmal RA, Lima JAC, Liu K, Olson J, Burke GL et al The relationship of left ventricular mass and geometry to incident cardiovascular events: the MESA (Multi-Ethnic Study of Atherosclerosis) study. J Am Coll Cardiol 2008;52:2148–55. 10.1016/j.jacc.2008.09.014 PMC2706368 19095132

[13] Zhang X, Cowan BR, Bluemke DA, Finn JP, Fonseca CG, Kadish AH et al Atlas-based quantification of cardiac remodeling due to myocardial infarction. PLoS One 2014;9:e110243. 10.1371/journal.pone.0110243 PMC4215861 25360520

[14] Buoso S, Joyce T, Schulthess N, Kozerke S. MRXCAT2.0: synthesis of realistic numerical phantoms by combining left-ventricular shape learning, biophysical simulations and tissue texture generation. J Cardiovasc Magn Reson 2023;25:25. 10.1186/s12968-023-00934-z PMC10116689 37076840

[15] Littlejohns TJ, Holliday J, Gibson LM, Garratt S, Oesingmann N, Alfaro-Almagro F et al The UK Biobank imaging enhancement of 100,000 participants: rationale, data collection, management and future directions. Nat Commun 2020;11:2624. 10.1038/s41467-020-15948-9 PMC7250878 32457287

[16] Bild DE, Bluemke DA, Burke GL, Detrano R, Diez Roux AV, Folsom AR et al Multi-ethnic study of atherosclerosis: objectives and design. Am J Epidemiol 2002;156:871–81. 10.1093/aje/kwf113 12397006

[17] Burns R, Dal Toso L, Mauger CA, Sojoudi A, Suinesiaputra A, Petersen SE et al Relationships between heart shape, function, and disease in 38,858 UK Biobank participants. J Cardiovasc Magn Reson 2025;27:101919. 10.1016/j.jocmr.2025.101919 PMC12780292 40467037

[18] Beetz M, Corral Acero J, Banerjee A, Eitel I, Zacur E, Lange T et al Interpretable cardiac anatomy modeling using variational mesh autoencoders. Front Cardiovasc Med 2022;9:983868. 10.3389/fcvm.2022.983868 PMC9813669 36620629

[19] Buoso S, Joyce T, Kozerke S. Personalising left-ventricular biophysical models of the heart using parametric physics-informed neural networks. Med Image Anal 2021;71:102066. 10.1016/j.media.2021.102066 33951597

[20] Qiao M, McGurk KA, Wang S, Matthews PM, O'Regan DP, Bai W. A personalized time-resolved 3D mesh generative model for unveiling normal heart dynamics. Nat Mach Intell 2025;7:800–11. 10.1038/s42256-025-01035-5 PMC12101970 40416329

[21] Moscoloni B, Beeche C, Chirinos JA, Segers P, Peirlinck M. Unveiling sex dimorphism in the healthy cardiac anatomy: fundamental differences between male and female heart shapes. J Physiol 2026;604:5512–34. 10.1113/JP288667 PMC13327772 40997328

[22] St Pierre SR, Peirlinck M, Kuhl E. Sex matters: a comprehensive comparison of female and male hearts. Front Physiol 2022;13:831179. 10.3389/fphys.2022.831179 PMC8980481 35392369

[23] Cheng S, Fernandes VR, Bluemke DA, McClelland RL, Kronmal RA, Lima JAC. Age-related left ventricular remodeling and associated risk for cardiovascular outcomes: the multi-ethnic study of atherosclerosis. Circ Cardiovasc Imaging 2009;2:191–8. 10.1161/CIRCIMAGING.108.819938 PMC2744970 19808592

[24] Fiechter M, Fuchs TA, Gebhard C, Stehli J, Klaeser B, Stahli BE et al Age-related normal structural and functional ventricular values in cardiac function assessed by magnetic resonance. BMC Med Imaging 2013;13:6. 10.1186/1471-2342-13-6 PMC3626771 23391039

[25] Pillai R, Zhang L, Peters K, Jha V, O'Donnell CJ, Manning WJ et al Age- and sex-differences and reference values for ventricular strain by cardiovascular magnetic resonance imaging in adults without cardiovascular disease or cardiovascular disease risk factors. J Cardiovasc Magn Reson 2025;27:101902. 10.1016/j.jocmr.2025.101902 PMC12138549 40300666

[26] Qiao MY, Basaran BD, Qiu HQ, Wang S, Guo Y, Wang YY et al Generative modelling of the ageing heart with cross-sectional imaging and clinical data. Lect Notes Comput Sc 2022;13593:3–12.

[27] Mauger CA, Ambale-Venkatesh B, Suinesiaputra A, Bluemke DA, Wu CO, Lima JAC et al Longitudinal trajectories of left ventricular myocardial remodeling: associations with cardiovascular risk factors in the multi-ethnic study of atherosclerosis. J Cardiovasc Magn Reson 2025;27:101943. 10.1016/j.jocmr.2025.101943 PMC12745149 40850633

[28] Eng J, McClelland RL, Gomes AS, Hundley WG, Cheng S, Wu CO et al Adverse left ventricular remodeling and age assessed with cardiac MR imaging: the multi-ethnic study of atherosclerosis. Radiology 2016;278:714–22. 10.1148/radiol.2015150982 PMC4770941 26485617

[29] Ugurlu D, Qian S, Fairweather E, Mauger C, Ruijsink B, Toso LD et al Cardiac digital twins at scale from MRI: open tools and representative models from ∼ 55000 UK Biobank participants. PLoS One 2025;20:e0327158. 10.1371/journal.pone.0327158 PMC12262899 40663525

[30] Isensee F, Jaeger PF, Kohl SAA, Petersen J, Maier-Hein KH. nnU-Net: a self-configuring method for deep learning-based biomedical image segmentation. Nat Methods 2021;18:203–11. 10.1038/s41592-020-01008-z 33288961

[31] Kabsch W . A solution for the best rotation to relate two sets of vectors. Acta Crystallographica Section A 1976;32:922–3.

[32] Joyce T, Buoso S, Stoeck CT, Kozerke S. Rapid inference of personalised left-ventricular meshes by deformation-based differentiable mesh voxelization. Med Image Anal 2022;79:102445. 10.1016/j.media.2022.102445 35468554

[33] Mannaerts HFJ, van der Heide JA, Kamp O, Stoel MG, Twisk J, Visser CA. Early identification of left ventricular remodelling after myocardial infarction, assessed by transthoracic 3D echocardiography. Eur Heart J 2004;25:680–7. 10.1016/j.ehj.2004.02.030 15084373

