# Supplementary material for: Sex-specific shape models to disentangle sex effects and age-associated anatomical variation in biventricular cardiac anatomy
Source: Eur Heart J Imaging Methods Pract. 2026 Sep 7;4(2):qyag156. doi: 10.1093/ehjimp/qyag156 (PMC13617352; doi:10.1093/ehjimp/qyag156)
Supplement: Supplementary file 1 [file qyag156_supplementary_data.docx]

# **Supplementary material**

***Figures***

**Figure A1.** Cosine similarity analysis between sex-agnostic and sex-specific PCA modes. top: cosine similarity between sex-specific male and female PCA modes, bottom: cosine similarity between sex-agnostic modes and sex-specific specific male (blue heatmap) and female (red heatmap) modes.

**Figure A2.** Anatomical variation across the first seven PCA modes (for ±4 standard deviations of each anatomical score). Bars show the percentage change from lower to higher scores for male-specific, female-specific, male sex-agnostic, and female sex-agnostic anatomical models. Metrics include LV blood pool volume, RV blood pool volume, myocardial mass, average LV wall thickness, and sphericity. Hatched bars indicate sex-agnostic models

**Figure A3**. Scatter plots of mode coefficients as a function of age, stratified by sex. Columns show Modes 1-5. The top and middle rows show sex-specific associations for males and females, respectively, while the bottom row shows sex-agnostic models with both sexes overlaid. Solid lines indicate fitted linear trends with shaded confidence intervals; points represent individual subjects.

***Tables***

**Table A1**. Demographic, anthropometric, haemodynamic, and clinical characteristics of the screened SwissHeart volunteers included in the study. Values are presented as mean (standard deviation) for age, weight, height, body surface area (BSA), systolic blood pressure (SBP), and diastolic blood pressure (DBP), and as number (%) for categorical variables*.*

| **Characteristics** | **Males**  (n = 185) | **Females**  (n = 187) |
| --- | --- | --- |
| **Demographics** |  |  |
| Age, years mean (SD) | 46 (15) | 46 (15) |
| Ethnicity, n (%) |  |  |
| Caucasian/White | 247 (96.5) | 233 (95.5) |
| Other | 9 (3.5) | 11 (4.5) |
|  |  |  |
| **Anthropometrics** |  |  |
| Weight, kg, mean (SD) | 81 (12) | 63 (9) |
| Height, cm mean (SD) | 180 (7) | 166 (7) |
| BSA, m^2^ mean (SD) | 2.01 (0.16) | 1.70 (0.12) |
|  |  |  |
| **Hemodynamic** |  |  |
| SBP, mmHg, mean (SD) | 132 (15) | 120 (16) |
| DBP, mmHg, mean (SD) | 80 (10) | 79 (11) |
|  |  |  |
| **Clinical characteristics** |  |  |
| No CV disease, n (%) | 193 () | 189 () |
| Bicuspid aortic valve, n (%) | 3 (1.2) | 0 (0.0) |
| Valve regurgitation, n (%) | 13 (5.1) | 14 (5.7) |
| Aortic aneurysm, n (%) | 9 (3.5) | 6 (2.5) |
| Other CV diseases, n (%) | 11 (4.3) | 11 (4.5) |
| Hypertension, n (%) | 27 (10.5) | 13 (5.3) |
| Diabetes, n (%) | 6 (2.3) | 0 (0.0) |
| Dyslipidaemia, n (%) | 150 (58.6) | 110 (45.1) |
| Smokers, n (%) | 11 (4.3) | 20 (8.2) |

**Table A2.** Age-stratified cardiac metrics for the male SwissHeart cohort. Values are presented as mean (standard deviation) for weight, height, BSA, systolic and diastolic blood pressures (SBP, DBP), left and right ventricular end-diastolic and end-systolic volumes (LVEDV, LVESV, RVEDV, RVESV), ejection fraction (LVEF, RVEF), LV myocardium volume (LVM), sphericity at end-diastole (Ψ_ED_), and mitral/tricuspid annular plane systolic excursion (MAPSE, TAPSE).

| **Biomarker** | | **Age groups** | | | | | |
| --- | --- | --- | --- | --- | --- | --- | --- |
|  |  | **18-29** | **30-39** | **40-49** | **50-59** | **60-69** | **70-85** |
| **N** | **-** | 49 | 51 | 45 | 47 | 41 | 17 |
| **Weight** | **kg** | 77(11) | 81(14) | 82(11) | 86(14) | 82(12) | 79(10) |
| **Height** | **cm** | 180(6) | 182(8) | 182(7) | 180(7) | 179(5) | 179(6) |
| **BSA** | **m^2** | 1.96(0.15) | 2.02(0.20) | 2.03(0.15) | 2.07(0.18) | 2.02(0.16) | 1.98(0.14) |
| **SBP** | **mmHg** | 127(12) | 127(12) | 128(13) | 135(17) | 138(15) | 148(17) |
| **DBP** | **mmHg** | 74(8) | 76(8) | 79(11) | 85(10) | 85(9) | 82(7) |
| **LVEDV** | **ml** | 149(21) | 154(26) | 141(23) | 137(24) | 130(25) | 128(25) |
| **LVESV** | **ml** | 61(13) | 62(13) | 55(11) | 55(15) | 52(12) | 48(10) |
| **LVEF** | **%** | 59(5) | 60(4) | 61(4) | 60(6) | 61(4) | 63(4) |
| **LVM** | **g** | 124(20) | 128(23) | 125(22) | 124(24) | 118(21) | 119(17) |
| **RVEDV** | **ml** | 149(24) | 152(24) | 141(26) | 139(27) | 132(29) | 131(25) |
| **RVESV** | **ml** | 62(15) | 62(15) | 56(16) | 56(14) | 55(15) | 51(10) |
| **RVEF** | **%** | 59(6) | 60(5) | 61(6) | 60(5) | 58(6) | 61(4) |
| $\boldsymbol{\Psi}_{\boldsymbol{ED}}$ | **-** | 0.25(0.04) | 0.25(0.04) | 0.24(0.05) | 0.25(0.04) | 0.26(0.05) | 0.25(0.06) |

**Table A3**. Age-stratified cardiac metrics for the female SwissHeart cohort. Values are presented as mean (standard deviation) for weight, height, BSA, systolic and diastolic blood pressures (SBP, DBP), left and right ventricular end-diastolic and end-systolic volumes (LVEDV, LVESV, RVEDV, RVESV), ejection fraction (LVEF, RVEF), LV myocardium volume (LVM), sphericity at end-diastole (Ψ_ED_), and mitral/tricuspid annular plane systolic excursion (MAPSE, TAPSE).

| **Biomarker** | | **Age groups** | | | | | |
| --- | --- | --- | --- | --- | --- | --- | --- |
|  |  | **18-29** | **30-39** | **40-49** | **50-59** | **60-69** | **70-85** |
| **N** | **-** | 42 | 45 | 46 | 45 | 40 | 18 |
| **Weight** | **kg** | 64(8) | 62(7) | 64(9) | 65(8) | 63(9) | 60(8) |
| **Height** | **cm** | 168(6) | 167(7) | 167(5) | 166(5) | 163(6) | 162(7) |
| **BSA** | **m^2** | 1.73(0.13) | 1.69(0.13) | 1.72(0.13) | 1.72(0.12) | 1.68(0.13) | 1.65(0.14) |
| **SBP** | **mmHg** | 116(11) | 113(12) | 113(12) | 121(13) | 130(17) | 144(17) |
| **DBP** | **mmHg** | 76(8) | 75(10) | 76(8) | 81(9) | 84(11) | 82(11) |
| **LVEDV** | **ml** | 115(17) | 110(16) | 102(15) | 97(11) | 91(14) | 84(15) |
| **LVESV** | **ml** | 44(7) | 41(7) | 37(8) | 34(5) | 33(7) | 27(7) |
| **LVEF** | **%** | 62(3) | 63(4) | 64(4) | 65(4) | 64(5) | 68(4) |
| **LVM** | **g** | 90(14) | 85(15) | 80(15) | 79(11) | 81(11) | 74(9) |
| **RVEDV** | **ml** | 111(19) | 104(18) | 94(16) | 93(13) | 87(17) | 78(14) |
| **RVESV** | **ml** | 44(11) | 39(10) | 32(9) | 32(7) | 30(8) | 26(7) |
| **RVEF** | **%** | 61(5) | 63(5) | 66(6) | 66(5) | 65(6) | 67(4) |
| $\boldsymbol{\Psi}_{\boldsymbol{ED}}$ | **-** | 0.23(0.05) | 0.25(0.04) | 0.24(0.04) | 0.26(0.05) | 0.25(0.05) | 0.25(0.05) |

### Breath-hold offsets correction

A 3D thorax scan was used as an anatomically consistent reference to correct for breath-hold–induced offsets in the cine acquisitions. The 3D volume was resampled to match the orientations of the short- and long-axis images. For each long-axis view, a stack of 20 slices with 1 mm thickness was generated, preserving the original plane normal. These respiratory-consistent stacks were temporally aligned with the cine acquisitions and segmented using the same nnU-Net models used for the cine images. Segmentation quality for long-axis views was assessed by computing the overlap with cine LV blood pools. If the overlap fell below 30%, short-axis labels were resampled into the long-axis plane to avoid propagation of segmentation errors.

Let $S_{k}^{\text{slice}}\in\mathbb{R}^{2}$ denote the segmentation mask of the $k$-th 2D slice of the short-axis cine, and let $R_{k}^{\text{ref}}\in\mathbb{R}^{2}$ denote the corresponding segmentations of the slice of the resliced 3D. The 2D in-plane rigid translation $\mathbf{t}_{k}\in\mathbb{R}^{2}$ was computed by minimizing the distance of the centroids of the LV blood pools. To limit excessive shifts due to noise, the displacement magnitude was capped:

$$\mathbf{t}_{k}\leftarrow\mathbf{t}_{k}\cdot min(1,\frac{d_{\text{max}}}{\parallel\mathbf{t}_{k}\parallel_{2}}),$$

where $d_{\text{max}}$ is the maximum allowed in-plane displacement in pixels.

For long-axis views, an additional through-plane correction was applied. Each cine slice $S_{k}^{\text{slice}}$ was aligned with every slice in the resampled stack using the in-plane translations, and the Dice similarity of the LV blood pool was computed. The through-plane translation $\mathbf{n}_{k}$was defined as the normal distance to the slice in the stack that maximized the Dice coefficient. The final corrected slice position incorporated both the in-plane translation $\mathbf{t}_{k}$ and the through-plane offset along the slice normal $\mathbf{n}_{k}$.

**Biventricular mesh fitting**

A template biventricular mesh (29) was rigidly aligned to each subject’s anatomy through a two-step similarity transformation. First, an initial landmark-based alignment was performed to account for global anatomical differences. Let

$$L_{m}={\{\boldsymbol{l}_{m}^{i}\}}_{i=1}^{N}\in\mathbb{R}^{3}, \text{ }L_{t}=\left\{ \boldsymbol{l}_{t}^{i} \right\}_{i=1}^{N} \in\mathbb{R}^{3},$$

be the sets of $N$ anatomical landmarks extracted from the mesh and target contours, respectively (LV apex and MV, TV and aortic landmarks). Each landmark set was mean-centred to remove translational effects:

$${\tilde{\boldsymbol{l}}}_{k}^{i}= \boldsymbol{l}_{k}^{i}- {\bar{\boldsymbol{l}}}_{k} \text{,} {\bar{\boldsymbol{l}}}_{k}= \frac{1}{N}\sum_{j=1}^{N} \boldsymbol{l}_{k}^{j} \text{for} k \text{in} \left\{ m,s \right\}.$$

A global isotropic scaling factor was computed from the Frobenius norms of the mean-centered matrices:

$$s= \frac{\left\| \tilde{L}_{t} \right\|_{F}}{\left\| \tilde{L}_{m} \right\|_{F}},\left\| \tilde{L}_{k} \right\|_{F}= \sqrt{\sum_{i=1}^{N} \left\| {\tilde{\boldsymbol{l}}}_{k}^{i} \right\|^{2}}\text{for} k \text{in} \left\{ m,s \right\},$$

After scaling, the optimal rigid rotation $\boldsymbol{R}$ was obtained by minimizing the least-squares alignment error (31) via SVD of the cross-covariance matrix $\boldsymbol{H}= \sum_{i=1}^{N} {\tilde{\boldsymbol{l}}}_{m}^{i}\left( {\tilde{\boldsymbol{l}}}_{t}^{i} \right)^{T}:$

$$\boldsymbol{R}=\boldsymbol{V}\boldsymbol{U}^{T}, \boldsymbol{H}=\boldsymbol{U}\boldsymbol{\Sigma}\boldsymbol{V} ,\det\left( \boldsymbol{R} \right)>0.$$

The translation vector was computed as:

$$\boldsymbol{t=}{\bar{\boldsymbol{l}}}_{t}\boldsymbol{-}s\boldsymbol{R}{\bar{\boldsymbol{l}}}_{m}\boldsymbol{,}$$

and the final mesh coordinates, $\boldsymbol{X}_{m}$, are obtained from the original template mesh coordinates, ${\hat{\boldsymbol{X}}}_{m},$as

$$\boldsymbol{X}_{m}=s\boldsymbol{R}{\hat{\boldsymbol{X}}}_{m}+\boldsymbol{t.}$$

After global similarity alignment, non-rigid refinement was performed using a free-form deformation (FFD) model defined over a regular control lattice enclosing the template mesh. Let

$$\mathcal{C=}\left\{ \mathbf{c}_{ijk}\in\mathbb{R}^{3} \right\},$$

denote a regular 3D lattice of control points spanning the bounding box of the current mesh configuration $\mathbf{X}_{m}$. Two lattices were used in a multi-resolution hierarchy: a coarse lattice, denoted with the subscript $c$ for global smooth deformations, and a fine lattice, denoted with the subscript $f$ for local geometric refinement allowed only in the plane of the short-axis. In the case of this work, the coarse lattice had 4 control points along each direction (4^3^ total points) and the finer one 8 (8^3^ total points). Each control point $\mathbf{c}_{ijk}^{g}$ was associated with a displacement vector $\mathbf{d}_{ijk}^{g}\in\mathbb{R}^{3}$ with $g \text{in} \{c,f\}$.

*Deformation model:* For each mesh vertex $\boldsymbol{X}_{m}$, the FFD displacement was defined as a weighted sum of neighbouring control-point displacements:

$$\mathbf{u}(\boldsymbol{X}_{m})=\sum_{g \in\{c,f\}} \sum_{\left( i,j,k)\in\mathcal{N(}p \right)} w_{ijk}^{g}(\boldsymbol{X}_{m})\text{ }\mathbf{d}_{ijk}^{g},$$

where $g$ indexes the FFD resolution level, $\mathcal{N(}p)$ denotes the set of control points influencing $\boldsymbol{X}_{m}$, $w_{ijk}^{g}(\boldsymbol{X}_{m})$ are precomputed trilinear basis weights satisfying

$$\sum_{\left( i,j,k)\in\mathcal{N(}p \right)} w_{ijk}^{g}\left( \boldsymbol{X}_{m} \right)=1 \forall g\in\{c,f\} .$$

The deformed mesh vertices were then given by:

$$\mathbf{X}_{m}^{'}=\mathbf{X}_{m}+\mathbf{u}\left( \mathbf{X}_{m} \right)= \mathbf{X}_{m}+\mathbf{u}_{c}\left( \mathbf{X}_{m} \right)+ \mathbf{u}_{f}\left( \mathbf{X}_{m} \right).$$

The unknowns of the fitting process were therefore the displacements $\mathbf{d}_{ijk}$of the control points of the two lattices. These were optimized minimizing a loss function composed of data consistency and regularization terms.

*Data consistency*: Geometric consistency with the observed contours $\mathbf{X}_{t}$ was enforced via a Chamfer distance between the deformed mesh surface and the target point sets:

$$\mathcal{L}_{\text{data}}=\text{chamfer}(\mathbf{X}_{m}^{'},\mathbf{X}_{t}),$$

Separate data terms were computed for each anatomical structure (LV endocardium, LV epicardium, RV endocardium, LV septum, great vessels, valves), and combined using structure-specific weights set to 1 for all LV and RV surfaces, to 0.1 for the great vessels and to 0.3 for the valves.

*FFD-level regularisation:* To ensure physically plausible deformations, the control-point displacements of both lattices were regularised using three complementary terms.

Excessive deformation was penalised with $\mathcal{L}_{\text{warp}}$ defined as

$$\mathcal{L}_{\text{warp}}=\sum_{g \in\{c,f\}} \frac{1}{N_{g}}\sum_{(i,j,k)} \parallel\mathbf{d}_{ijk}\parallel^{2},$$

where $N_{g}$ was the total number of control points of each lattice. In this work, the coarse and fine contributions were weighted with ${10}^{-4}$ and ${2\cdot10}^{-2}$.

Smoothness and regularity of the deformation fields of the lattice grids were enforced with the contribution of a $\mathcal{L}_{\text{smooth}}$ and $\mathcal{L}_{\text{bend}}.$The first loss penalised first-order finite differences between neighbouring control points:

$$\mathcal{L}_{\text{smooth}}= \sum_{g \in\{c,f\}} \frac{1}{N_{g}}\sum_{i,j,k} \left( \left\| c_{i+1,j,k}-c_{i,j,k} \right\|^{2}+\left\| c_{i,j+1,k}-c_{i,j,k} \right\|^{2}{+ \left\| c_{i,j,k+1}-c_{i,j,k} \right\|}^{2} \right) .$$

The coarse and fine contributions were weighted with ${10}^{-3}$ and ${2\cdot10}^{-2}$. The second loss was used to suppress oscillatory or non-physical deformations

$$\mathcal{L}_{\text{bend}}=\sum_{g \in\{c,f\}} \frac{1}{N_{g}^{'}}\sum_{i,j,k} \left\| c_{i+1,j,k}+c_{i-1,j,k}+ c_{i,j+1,k}+c_{i,j-1,k}+ c_{i,j,k+1}+c_{i,j,k-1}-6c_{i,j,k} \right\|^{2},$$

where ${N'}_{g}$ is the total number of internal lattice points where the loss can be defined. For this loss, the coarse and fine contributions were weighted with ${10}^{-6}$ and ${2\cdot10}^{-2}$.

*Mesh-level regularization*: Due to the sparsity of the target control points, the fine-scale driven displacement $\mathbf{u}_{f}$ of the mesh points is strongly discouraged to have a component in the normal direction to the short-axis acquisition, $\mathbf{n}$, with the loss defined over the total $N_{m}$ mesh points as

$$\mathcal{L}_{\text{normal}}=\frac{1}{N_{m}}\sum_{j=1}^{N_{m}} \left\| \boldsymbol{u}_{f}^{i}\cdot\mathbf{n} \right\|^{2}.$$

Although deformation is parameterised via FFD, the uniform Laplacian smoothing, $\mathcal{L}_{\mathrm{laplacian}},$ was used to penalize non-smooth mesh vertex displacements and $\mathcal{L}_{\mathrm{vel}}$was used to enforce inter-frame velocity penalization. Specifically,

$$\mathcal{L}_{laplacian =}\frac{1}{\sum_{a=1}^{N_{A}} K_{A}}{\sum_{a=1}^{N_{A}} \sum_{m=1}^{K_{A}} \left\| \boldsymbol{u}\left( \boldsymbol{X}_{m} \right)-\frac{1}{|N(m)|}\sum_{j\in\mathcal{N}\left( m \right)} \boldsymbol{u}\left( \boldsymbol{X}_{j} \right) \right\|}^{2},$$

where $m$ is one of the $K_{A}$ points of the $N_{A}$ anatomical region and $N_{j}$ are the neighbouring points of the $m$-th point.

Finally, temporal continuity was ensured through

$$\mathcal{L}_{\mathrm{vel}}=\frac{1}{N_{m}}\sum_{i=1}^{N_{m}} \left( \boldsymbol{u}_{m}^{t}-{\boldsymbol{u}^{t-1}}_{m} \right)$$

where, $t$ and $t-1$ are two consecutive frames of the cardiac cycle.
The total objective function is obtained by the weighted summation of all losses. The weights are manually optimised and kept constant on the full SwissHeart dataset. The problem was minimized using the Adam optimizer over approximately 200 iterations per frame. All computations were implemented in GPU-accelerated PyTorch, with coordinate normalization and configurable loss weighting to ensure numerical stability and allow for per-patient refinement in challenging anatomies.

### Method verification

The fully automated segmentation and mesh fitting pipeline was successfully applied to the entire cohort. After volumetric and strain quality control, 10 volunteers were excluded, resulting in a 98% overall success rate.

Segmentation accuracy was assessed against manual delineations. In short-axis views of 158 subjects, Dice similarity coefficients were 0.95 (0.03) for the LV blood pool, 0.90 (0.04) for the RV blood pool, and 0.82 (0.05) for the LV myocardium. LV myocardium dice scores of 102 subjects were 0.84 (0.09) in the two-chamber view, and 0.84 (0.07) in the three-chamber view, and dice scores for LV myocardium and RV blood pool were 0.95 (0.02) and 0.82 (0.12) in the four-chamber view.

Volume agreement was further evaluated in 160 subjects. Mean differences between automated and manual segmentations were 0.3 (7) mL for LVEDV and 0.1 (15) mL for LVESV. RV volumes showed slightly larger biases of −10 (17) mL for RVEDV and −9 (24) mL for RVESV. Across the full cohort, mesh-derived volumes closely matched nnU-Net estimates, with errors of 7 (16) mL and 17 (18) mL for LVEDV and LVESV, and −12 (12) mL and −9 (18) mL for the corresponding RV volumes.

Breath-hold correction displacements averaged 4 (5) pixels for short-axis stacks and 7 (8) pixels for long-axis views.
